# Prevalence and Risk Factors of Surgical Treatment for Klippel–Feil Syndrome

**DOI:** 10.3389/fsurg.2022.885989

**Published:** 2022-06-07

**Authors:** Linyao Ding, Xin Wang, Yu Sun, Fengshan Zhang, Shengfa Pan, Xin Chen, Yinze Diao, Yanbin Zhao, Tian Xia, Weishi Li, Feifei Zhou

**Affiliations:** ^1^ Key Laboratory of Spinal Disease Research, Department of Orthopedic Surgery, Peking University Third Hospital, Peking University, Beijing, China; ^2^ Department of Orthopedic Surgery, The Affiliated Hospital of Yunnan University, Yunnan University, Kunming, China

**Keywords:** Klippel–Feil syndrome, surgical treatment, prevalence, risk factors, Samartzis classification

## Abstract

**Background:**

Recently, there have been some reports on surgical treatment for Klippel–Feil syndrome, but the prevalence and risk factors of surgery have not been well evaluated. This study sought to find the prevalence and potential risk factors of surgical treatment.

**Methods:**

A retrospective radiographic review of 718 Klippel–Feil syndrome patients seen at Peking University Third Hospital from January 2010 to October 2017 was performed. Parameters included age, gender, deformity, cervical instability, Samartzis classification, and surgical treatment. Based on the surgical treatment they received, patients were divided into a surgery group and a non-surgery group. Prevalence and possible risk factors of surgical treatment were assessed.

**Results:**

A total of 718 Klippel–Feil syndrome patients, including 327 men and 391 women, with an average age of 46.8 years were enrolled. According to the Samartzis classification scheme, 621 cases (86.5%) were classified as type I, 48 cases (6.7%) were classified as type II, and 49 cases (6.8%) were classified as type III, respectively. The most commonly fused segments were C2–3 (54.9%) and C5–6 (9.3%). Of all 718 patients, 133 (18.5%) patients underwent surgical treatment, mainly via the posterior approach (69.9%). The clinical factors included age, gender, deformity, instability, and Samartzis classification. Men were more likely to require surgical treatment (*p* < 0.001). Patients with instability (*p* < 0.001) or patients with deformity (*p* = 0.004) were also more likely to undergo surgery. All three of these variables were included in the binary regression analysis. Finally, gender (*p* < 0.001) and unstable joints (*p* < 0.001) were identified to be independently associated with surgical treatment. Gender was the most important risk factor with men being 2.39 times more likely to have surgical treatment, while patients with instability were 2.31 times more likely to receive surgery.

**Conclusion:**

The prevalence of patients with Klippel–Feil syndrome requiring surgery was 18.5%, with the majority undergoing posterior cervical surgery. Gender and instability were indemnified as independent risk factors leading to surgical treatment.

## Introduction

Klippel–Feil syndrome (KFS) is a rare congenital cervical fusion condition, first described by Maurice Klippel and Andre Feil in 1912 (1). It is characterized by the congenital fusion of ≥2 cervical vertebrae, resulting from abnormal embryonic development during the first 3–8 weeks of gestation (2). The typical clinical triad of a low posterior hairline, a short neck, and restricted neck motion was originally recognized as the hallmark of KFS (3, 4), but numerous spinal and extraspinal anomalies have now been documented in KFS patients (5–8).

The global incidence of KFS is estimated to be 0.71% (9, 10), and the condition mainly affects female individuals (60% cases). However, the exact prevalence of this condition has not been well assessed due to the fact that asymptomatic patients without an obvious physical deformity are often not found to have KFS until a clinical event such as trauma requires cervical spine imaging (3, 11, 12). Therefore, the usage of radiology remains an important way to detect and evaluate KFS.

The fusion patterns of KFS patients are widely classified as single fusion, multi-continuous fusion, and multi-non-continuous fusion, as defined in the Samartzis classification (13). Patients with persistent myelopathy or radiculopathy, instability, or deformity warrant consideration for surgical treatment (14, 15). Our aim was to identify the factors leading to surgery for KFS patients and to validate the prevalence of KFS based on the Samartzis classification.

## Materials and Methods

### Subjects and Data

This study was a retrospective radiographic review of KFS patients at Peking University Third Hospital, Beijing, China. After receiving Institutional Review Board approval from Peking University Third Hospital (IRB no:160-02), we searched our radiographic system using the key term “cervical fused segments” and found 1,014 patients with cervical fused segments, 296 of whom were subsequently excluded. Of these excluded patients, 86 patients did not have congenitally fused cervical segments, 181 patients only had an occipital fusion, and 12 patients only had a fusion between C7 and thoracic vertebra, while 17 patients lacked demographic information. We finally enrolled 718 consecutive KFS patients with basic information and complete radiographic records seen from January 2010 to October 2017 for the analysis.

### Parameters and Variables

**Figure 1:**
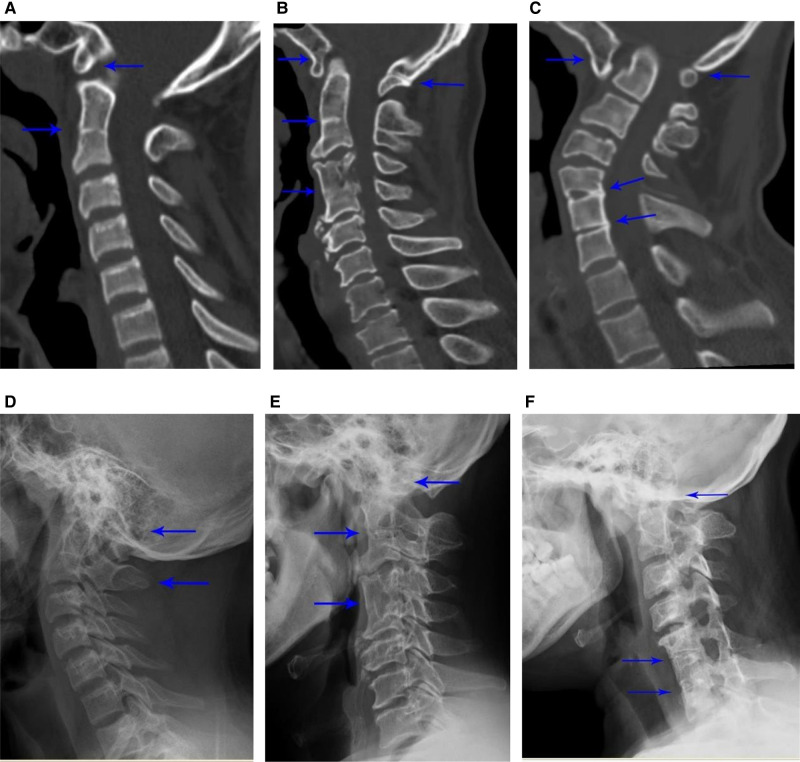
Patients with Klippel–Feil syndrome (KFS) and occipital fusion. Examples of different types of KFS patients with occipital fusion: type I – (**A**) computed tomography (CT) scan and (**D**) radiograph showing C2–3 single fusion with occipital fusion; type II – (**B**) CT scan and (**E**) radiograph showing noncontiguous levels of fusion (C2–3 and C4–5) with occipital fusion; type III – (**C**) CT scan and (**F**) radiograph showing three levels of contiguous fusion at C5-7 with occipital fusion. The site of fusions is indicated by *arrows*.

Radiographs collected during the evaluation of KFS patients entailed cervical lateral, anteroposterior (AP), extension, and flexion views along with computed tomography (CT) scanning as well as magnetic resonance imaging (MRI) of the cervical spine. Radiographs were initially used to verify diagnoses and then reviewed to identify congenitally fused segments, which were defined as congenital bone bridges between the adjoining vertebrae or posterior elements without movement during flexion and extension. Each patient’s age and gender were obtained, and their clinical history with presenting symptoms and the use of surgical or non-surgical treatment were analyzed. When surgical treatment was performed, the approach of the surgery was noted and collected. All radiographic evaluations were performed by an orthopedic spine surgeon, and crucial radiographic parameters consisting of the number of congenitally fused segments (C1–7), whether the patient had a single fusion or multiple fusions, and the presence of atlantooccipital fusion were also evaluated. Each radiograph was classified using the Samartzis classification scheme (Figure 1).

The instability of cervical segments was evaluated according to previously defined metrics. Patients with neck pain, headache, or neurological signs should be screened for cervical spine instability. Atlantoaxial instability (AAI) was defined as an anterior atlantodental interval (ADI) of >3 mm. Subaxial translation in the C3–7 vertebrae (SAS) was defined as a 2-mm translation of horizontal displacement of a single vertebra in relation to an adjacent vertebra. Additionally, the following types of deformity were considered: musculoskeletal deformity (defects of cervical vertebral formation, defects of thoracic vertebrae, and abnormalities in alignment), neural deformity (basilar invagination and Chiari malformation), and other deformities (torticollis). Moreover, information about whether the patient received surgical treatment was collected, and the surgical approach (e.g., anterior, posterior, or both) was recorded. The indications of surgery were radiculopathy or myelopathy symptoms, spinal cord compression, and instability of the cervical spine (mainly AAI). Surgery type and approach were decided upon by individual surgeons.

### Statistical Analysis

Statistical analysis was performed using SPSS version 26.0 (IBM Corporation, Armonk, NY, USA). Initial descriptive and frequency statistics were conducted. A chi-squared analysis of categorical variance was conducted, and Fisher’s exact test was considered when the cell count was less than 5. An appropriate *t*-test, correlation test analysis, parametricity analysis, and logistic regression modeling were performed. All statistical testing was two-sided. Statistical significance was established as *p* < 0.05.

## Results

### Population

A total of 718 KFS patients, including 327 men (45.5%) and 391 women (54.5%), were enrolled in this study. The mean age of the study participants was 46.8 years old (range, 4–92 years; standard deviation, 16.986 years). The most common level of congenitally fused segments was C2–3 (54.9%, 134 cases were C2–3 with atlantooccipital fusion) followed by C5–6 (9.2%) and C3–4 (8.5%). Additional frequencies of fusion levels are demonstrated in Table 1. With regards to the Samartzis classification, 621 patients (86.5%) were classified as type I, 48 patients (6.7%) were classified as type II, and 49 patients (6.8%) were classified as type III cases. Stratification of the classification type by gender was also performed (Table 2).

**Table 1:** Distribution of congenital fusion levels.

Samartzis classification	Congenital fusion level	Frequency (%)
Type I	O–C1 with C1–2	2 (0.3%)
	C1–2	2 (0.3%)
	O–C1 with C2–3	134 (18.7%)
	C2–3	260 (36.2%)
	O–C1 with C3–4	1 (0.1%)
	C3–4	60 (8.4%)
	O–C1 with C4–5	2 (0.3%)
	C4–5	57 (7.9%)
	C5–6	67 (9.3%)
	O–C1 with C6–7	1 (0.1%)
	C6–7	35 (4.9%)
Type II	O–C1 with multi	14 (1.9%)
	Multi	34 (4.7%)
Type III	O–C1 with multi	3 (0.4%)
	Multi	46 (6.4%)

**Table 2:** The incidence of Klippel–Feil syndrome (KFS) classification types to gender.

Samartzis classification	Male	Female
Type I	283 (45.6%)	338 (54.4%)
Type II	22 (45.8%)	26 (54.2%)
Type III	22 (44.9%)	27 (55.1%)

### Surgical Treatment

Of the 718 enrolled KFS patients, 133 (18.5%) received surgical treatment, including 82 men (61.7%) and 51 women (38.3%). In total, 17.7% type I patients, 27.1% type II patients, and 20.4% type III patients were treated with surgery. Percentages of fusion levels managed with surgical treatment are shown in Table 3.

**Table 3:** The distribution of surgical treatment in fusion levels.

Samartzis classification	Congenital fusion level	Surgery (Total)	Percentage
Type I	C1–2 with O–C1	1 (2)	50%
	C1–2	1 (2)	50%
	C2–3 with O–C1	28 (134)	20.9%
	C2–3	29 (260)	11.1%
	C3–4 with O–C1	0 (1)	0
	C3–4	14 (60)	23.3%
	C4–5 with O–C1	0 (2)	0
	C4–5	13 (57)	22.8%
	C5–6	17 (66)	25.8%
	C6–7 with O–C1	1 (1)	100%
	C6–7	6 (35)	17.1%
Type II	Multi with O–C1	7 (14)	50%
	Multi	6 (34)	17.6%
Type III	Multi with O–C1	1 (3)	33.3%
	Multi	9 (46)	19.6%

We also recorded the surgical approach (e.g., anterior, posterior, or both) of KFS patients who had undergone surgical treatment. The results showed that 33 patients (24.8%) received the anterior approach, 93 patients (69.9%) were treated through the posterior approach, and 7 patients (5.3%) underwent surgery using both approaches (Table 4). The distribution of various indications for surgical approaches is shown in Table 5

**Table 4:** The distribution of the type of surgery.

Samartzis classification	Type of surgery		
	Anterior	Posterior	Both
Type I	29 (26.4%)	78 (70.9%)	3 (2.7%)
Type II	2 (15.4%)	9 (69.2%)	2 (15.4%)
Type III	2 (20.0%)	6 (60.0%)	2 (20.0%)

**Table 5:** The distribution of various indications for surgery approaches.

Indication	Type of surgery		
	Anterior	Posterior	Both
Deformity			
Yes	6 (14.6%)	30 (73.2%)	5 (12.2%)
No	27 (29.3%)	63 (68.5%)	2 (2.2%)
Instability			
Yes	8 (17.8%)	34 (75.6%)	3 (6.7%)
No	25 (28.4%)	59 (67.0%)	4 (4.5%)

### Risk Factors

In all, 133 of the 718 KFS patients underwent surgical treatment. The clinical factors we acquired included age, gender, deformity, instability, and Samartzis classification. The comparisons and evaluation of parameters are summarized in Table 6. Male patients (*p *< 0.001), instability (*p *< 0.001), and deformity (*p *= 0.004) were factors that increased the chance of receiving surgery.

**Table 6:** Comparison of patients with or without surgical treatment.

Variable	Surgery (*n *= 133)	Non-surgery (*n *= 585)	*p*-value
Mean age (year)	47.24 ± 17.65	46.70 ± 16.85	0.741^a^
Age (year)			0.716^b^
<60	105 (78.9%)	470 (80.3%)	
≥60	28 (21.1%)	115 (19.7%)	
Gender			<0.001^b^
Male	82 (61.7%)	245 (41.9%)	
Female	51 (38.3%)	340 (58.1%)	
Deformity			0.004^b^
Yes	41 (30.8%)	113 (19.3%)	
No	92 (69.2%)	472 (80.7%)	
Instability			<0.001^b^
Yes	45 (33.8%)	100 (17.1%)	
No	88 (66.2%)	485 (82.9%)	
Samartzis classification			0.257^b^
Type I	110 (82.7%)	511 (87.4%)	
Type II	13 (9.8%)	35 (6.0%)	
Type III	10 (7.5%)	39 (6.7%)	

^a^ *p-value by t-test.*

^b^ *p-value by χ^2^*.

All three variables (gender, instability, and deformity) were included in the binary logistic regression analysis (Table 7). Gender (*p *< 0.001) and unstable joint (*p *< 0.001) were finally identified to be independently associated with surgical treatment. Gender was the most crucial risk factor with men being 2.39 times more likely to undergo surgical treatment, while KFS patients with instability were 2.31 times more likely to undergo surgery. The deformity was not confirmed as a potential independent risk factor for surgical treatment.

**Table 7:** Result of logistic regression.

Variable	OR	95% CI	*p-*value
Gender (male)	2.392	1.611, 3.522	<0.001
Deformity (yes)	1.470	0.920, 2.348	0.107
Instability (yes)	2.312	1.458, 3.668	<0.001

*Abbreviations: OR, odds ratio; CI, confidence interval.*

## Discussion

KFS is a rare disease, characterized as a condition of congenital fusion of at least two cervical vertebrae. The diagnosis of KFS is usually made based on clinical features and radiographic evaluations including plain radiographs (X-rays), CT, and MRI. However, the clinical triad of KFS is found in less than 50% KFS patients, and recent studies have shown that most sporadic KFS cases are identified by the radiographic evaluation performed incidentally as a lot of cases have asymptomatic single fused cervical segments (11, 16).

In this study, all radiographs of KFS patients treated at Peking University Third Hospital between January 2010 and October 2017 were evaluated. Of the 718 KFS patients that met the study inclusion criteria, 45.5% cases involved male patients, and 54.5% involved female patients. The most common levels of congenitally fused segments were C2–3 (54.9%) and C5–6 (9.2%), coinciding with the findings of previous literature. Further, there were 621 type I, 48 type II, and 49 type III cases, and it was clear that type I (86.5%) was the most common Samartzis classification in our study.

Regarding the surgical treatment, we found that 133 of the 718 (18.5%) enrolled KFS patients were treated by surgery, including 82 men (61.7%) and 51 women (38.3%). Of them, 33 patients (24.8%) took the anterior approach, 93 patients (69.9%) received surgery via the posterior approach, and 7 patients (5.3%) were treated using both approaches.

We evaluated the parameters to find possible risk factors. It seemed that gender, instability, and deformity were associated with KFS surgical treatment. Following the binary logistic regression analysis, gender and instability were identified as independent risk factors. Gender was the most crucial risk factor with men being 2.39 times more likely to undergo surgical treatment, while KFS patients with instability were 2.31 times more likely to receive surgery. However, we could not find any clear direct relationship between gender and surgery, so more studies are needed to demonstrate this.

The surgical treatment of patients with KFS presenting with radiculopathy or myelopathy manifestations, deformity, or definite instability is necessary (14, 15). In this context, anterior surgical treatment includes anterior cervical discectomy and fusion and anterior cervical corpectomy and fusion (17), while posterior surgery mainly consists of decompression and fusion surgical treatment (18). Emergency fusion surgery should be considered when the patients present with an unstable cervical spine, such as with the presence of foramen magnum occipitalis stenosis, basal ganglia depression, or any radiological evidence of progressive cervical instability.

Our study validated the previous study by Samartzis et al. (13), who reported an increased frequency of KFS in women (Table 2). However, we found that surgical treatment did not appear to be more prevalent in any single type of KFS. Our study demonstrated that up to 75.6% patients may undergo posterior fusion with laminectomy to address instability and myelopathy.

There were 145 patients (20.2%) diagnosed with instability in our report, and 31% of them underwent the surgery. The report from Samartzis et al. (19) demonstrated that a thorough evaluation given a high risk of cervical instability (cranial subluxation or atlantoaxial rotational subluxation) is necessary for patients with KFS. In their study, KFS patients were treated with surgery according to their instability status. This is consistent with our finding that instability is a potential independent risk factor for surgical treatment.

Nouri et al. (20) conducted a study that included the imaging data and clinical characteristics of 592 patients, including 14 diagnosed with KFS. They found that the center of the cervical spine was closely associated with spinal cord compression; spinal cord compression was found below the level of C3/4 fusion and above (9/10), while the compression was found above the plane of C5/6 fusion and below (8/8). As for the fusion level of C4/5, spinal cord compression was found in adjacent segments above and below. Surgery for myelopathy seemed to occur at center levels of the cervical spine, but the Samartzis classification, which categorizes different fusion levels of cervical segments, was found to have nothing to do with the decision to proceed to surgical treatment (*p *= 0.257).

Regarding spinal deformity, 154 patients in our study had a deformity, and 26.6% of them finally underwent surgery. Zhou et al. (21) suggested that patients with KFS tend to have spinal deformities and non-neurological deformities, and more KFS patients chose to undergo decompression surgery than not in their study. Hachem et al. (22) defined KFS patient phenotypes that were associated with surgical intervention through the principal component analysis and proposed that cervical spine surgery was associated with axial cervical spine anomalies and cervical subluxation, while cranial surgery had a high association with Chiari malformation. We were unable to find a specific relationship of deformity in the absence of Chiari malformation that showed an increased requirement for surgical intervention.

Limitations to our study include its retrospective nature, whereby patients who already showed symptoms were referred to our institution leading to a greater prevalence of surgical intervention in this study. The choice for surgery was also made by several surgeons, thereby leading to the heterogeneity of surgical treatment. Although we enrolled a large sample of patients who were diagnosed with KFS, most cases involved C2–3 fusion, which would make it hard to identify the correlation between risk factors and KFS prevalence. Further studies are warranted to find the exact risk factors of KFS surgical treatment and to test the exact correlation between deformity and KFS surgical treatment.

## Conclusion

We present a large analysis of KFS patients and their receipt of surgical treatment. In our study, the most prevalent pattern was Samartzis type I, defined as a single congenitally fused cervical segment, and the most common level of congenitally fused segments was C2–3. With regards to surgical treatments, the prevalence was 18.5%, and most operations were conducted via the posterior approach. However, gender and instability were identified as independent risk factors associated with surgical treatment. Gender was the most crucial risk factor with men being 2.39 times more likely to undergo surgical treatment than women, and KFS patients with instability were 2.31 times more likely to receive surgery. However, the deformity was not proven to be a potential independent risk factor for surgical treatment.

## Data Availability

The raw data supporting the conclusions of this article will be made available by the authors, without undue reservation.
